# Telehealth Delivery of Group Format Cognitive Rehabilitation to Older Veterans With TBI

**DOI:** 10.1093/geroni/igab046.2177

**Published:** 2021-12-17

**Authors:** Erica Kornblith, Sara Schweizer, Kristine Yaffe, Tatjana Novakovic-Agopain

**Affiliations:** 1 SFVA/UCSF, San Francisco, California, United States; 2 SFVA, San Francisco, California, United States; 3 UCSF, UCSF, California, United States

## Abstract

Traumatic brain injury (TBI) is common among older adults, with significant public health costs, and advanced age is a risk factor for poor outcomes after TBI. Older Veterans with TBI-related cognitive and emotional dysfunction without dementia may benefit from cognitive rehabilitation, particularly executive function training, and technology may promote optimal functioning for these patients by increasing access to such treatments. Dr. Kornblith will present pilot data on one such promising group intervention, Goal-Oriented Attentional Self-Regulation (GOALS), administered via in-home video telehealth. Themes gleaned from qualitative feedback collected throughout the intervention and post-treatment feedback questionnaires include the importance of communication and a smooth process with clear instructions for joining study sessions. Preliminary data suggest that only minor adaptions to the existing GOALS protocol are required for telehealth delivery and that delivering group-based executive function training to older TBI-exposed older Veterans with cognitive complaints via telehealth is feasible and acceptable.

